# Effectiveness of educational outreach in infectious diseases management: a cluster randomized trial in Uganda

**DOI:** 10.1186/s12889-016-3375-4

**Published:** 2016-08-04

**Authors:** Martin Kayitale Mbonye, Sarah M. Burnett, Sarah Naikoba, Allan Ronald, Robert Colebunders, Jean-Pierre Van Geertruyden, Marcia R. Weaver

**Affiliations:** 1 Infectious Diseases Institute, College of Health Sciences, Makerere University, Kampala, Uganda; 2 School of Statistics and Planning, College of Business and Management Sciences, Makerere University, Kampala, Uganda; 3 Global Health Institute, Faculty of Medicine and Health Sciences, University of Antwerp, Antwerp, Belgium; 4 Accordia Global Health Foundation, Washington, DC USA; 5 PATH, Washington, DC USA; 6 Save the Children, Kampala, Uganda; 7 Department of Medicine, University of Manitoba, Winnipeg, Manitoba Canada; 8 Department of Clinical Sciences, Institute of Tropical Medicine, Antwerp, Belgium; 9 Department of Global Health, University of Washington, International Training and Education Center for Health (I-TECH), Seattle, WA USA; 10 Department of Global Health, University of Washington, Institute for Health Metrics and Evaluation, Seattle, WA USA

**Keywords:** Educational outreach, Classroom training, Mid-level health providers, Uganda

## Abstract

**Background:**

Integrated Infectious Diseases Capacity Building Evaluation (IDCAP) teams designed and implemented two health worker in-service training approaches: 1) an off-site classroom-based integrated management of infectious diseases (IMID) course with distance learning aspects, and 2) on-site support (OSS), an educational outreach intervention. We tested the effects of OSS on workload and 12 facility performance indicators for emergency triage assessment and treatment, HIV testing, and malaria and pneumonia case management among outpatients by two subgroups: 1) mid-level practitioners (MLP) who attended IMID training (IMID-MLP) and 2) health workers who did not (No-IMID).

**Methods:**

Thirty-six health facilities participated in the IDCAP trial, with 18 randomly assigned to Arm A and 18 to Arm B. Two MLP in both arms received IMID. All providers at Arm A facilities received nine monthly OSS visits from April to December 2010 while Arm B did not. From November 2009 to December 2010, 777,667 outpatient visits occurred. We analyzed 669,580 (86.1 %) outpatient visits, where provider cadre was reported. Treatment was provided by 64 IMID-MLP and 1,515 No-IMID providers. The effect of OSS was measured by the difference in pre/post changes across arms after controlling for covariates (adjusted ratio of relative risks = a RRR).

**Results:**

The effect of OSS on patients-per-provider-per-day (workload) among IMID-MLP (aRRR = 1.21; *p* = 0.48) and No-IMID (aRRR = 0.90; *p* = 0.44) was not statistically significant. Among IMID-MLP, OSS was effective for three indicators: malaria cases receiving an appropriate antimalarial (aRRR = 1.26, 99 % CI = 1.02-1.56), patients with negative malaria test result prescribed an antimalarial (aRRR = 0.49, 99 % CI = 0.26-0.92), and patients with acid-fast bacilli smear negative result receiving empiric treatment for acute respiratory infection (aRRR = 2.04, 99 % CI = 1.06-3.94). Among No-IMID, OSS was effective for two indicators: emergency and priority patients admitted, detained or referred (aRRR = 2.12, 99 % CI = 1.05-4.28) and emergency patients receiving at least one appropriate treatment (aRRR = 1.98, 99 % CI = 1.21-3.24).

**Conclusion:**

Effects of OSS on workload were not statistically significant. Significant OSS effects on facility performance across subgroups were heterogeneous. OSS supported MLP who diagnosed and treated patients to apply IMID knowledge. For other providers, OSS supported team work to manage emergency patients. This evidence on OSS effectiveness could inform interventions to improve health workers’ capacity to deliver better quality infectious diseases care.

**Electronic supplementary material:**

The online version of this article 10.1186/s12889-016-3375-4) contains supplementary material, which is available to authorized users.

## Background

Sub Saharan Africa experiences 37 % of the total deaths attributable to infectious diseases globally [1], but contains only 3 % of the world’s health workers [2]. The shortage of physicians in sub Saharan Africa is at critical levels, with only 2 physicians per 10,000 population compared to 28 in high-income regions [3]. This shortage of physicians has led to many physician roles, such as initiation of antiretroviral therapy shifting to mid-level practitioners (MLP) with fewer years of medical training [4], formally [5–7] and informally [8, 9]. Yet as reported in one Ugandan study, many of these MLP have not been trained to take on these tasks [10]. They therefore require capacity building to meet the demands of changing health policies and guidelines [11]. Some studies have shown that with additional training MLP can competently execute physicians’ roles and, in some instances, match their performance [12–14]. However, most trainings for MLP are classroom-based, often taking them away from their workstations [15, 16], disrupting workflows and limiting patients’ access to care. Several systematic reviews show that classroom-based training may not equip the attendants with sufficient skills to ensure adherence to clinical guidelines [17–19].

There is increasing interest from experts and donors in favor of educational outreach and continuous quality improvement (CQI) approaches [20–24] that integrate across diseases [25–28]. Facility-based educational outreach trainings increase opportunities for team-based interactions during MLP learning [20, 29] and reduces MLP time away from health facilities. Several studies have shown that educational outreach both with and without CQI activities can improve the quality of patient care [5, 30–35]. It may also build capacity in patient care for almost all providers, without increasing workload on the MLP who do not attend trainings.

Between 2009 and 2010, the curriculum development and mobile teams of the Integrated Infectious Diseases Capacity Building Evaluation (IDCAP) developed and delivered two novel interventions to build capacity for treatment and prevention of infectious diseases: 1) the Integrated Management of Infectious Disease (IMID) course with distance learning aspects, taught at the Infectious Diseases Institute in Kampala, Uganda, and 2) facility-based on-site support (OSS) visits, an educational outreach intervention with CQI activities [29]. Implemented as a mixed design with pre/post and cluster randomized trial components, IDCAP tested the effect of IMID training and OSS on individual clinician competence [36], practice [37], mortality among children less than five years of age [20, 38], and facility performance [33, 39]. IMID was associated with a statistically significant 9.8 % absolute increase in clinical competence, whereas OSS was not associated with an incremental improvement in the same outcomes. [36]. For clinical practice, IMID was associated with statistically significant improvements in taking a patient’s history and physical examination, and OSS was associated with an incremental improvement in these tasks [37]. Child mortality increased in both arms, but the incremental effect of OSS delayed the increase during the intervention [38].

In the main trial analysis of facility performance, [33] *Weaver* et al. reported the effects of IDCAP’s IMID and OSS on 23 facility performance indicators. Facility performance refers to the process of care, and may reflect the contributions of more than one provider at a facility. For example, the proportion of malaria suspects with a malaria test result recorded (Indicator 4), reflects whether or not the clinician ordered the test, and the laboratory professional performed the test and recorded the result. Of the 23 indicators, 12 were based on a data surveillance system with data on each outpatient visit. The other 11 indicators were based on Ministry of Health Registers for HIV care, maternal, child and newborn health and tuberculosis. The 12 indicators based on the data surveillance system are presented in Table 1, and include three indicators for emergency triage assessment and treatment, four indicators for malaria case management, four indicators for case management of respiratory illness, and one indicator for HIV testing.

**Table 1:** Definitions of facility performance indicators

Program area and performance indicator		Definition	Reference
Emergency Triage, Assessment and Treatment (ETAT)			
1	Proportion of outpatients triaged	Numerator: Number of outpatients triaged, meaning that the patient was classified as emergency, priority, or queue, or an emergency sign was noted in the triage section of the form. Denominator: Number of outpatients	[50, 51]
2	Proportion of emergency and priority patients who were admitted, detained or referred	Numerator: Number of emergency and priority patients admitted, detained or referred for care. Denominator: Number of outpatients classified as emergency or priority or an emergency sign was noted in the triage section of the form. In line with World Health Organization guidelines, emergencies were defined as patients with one or more of the ABCDO triage signs (i.e., Airway; Breathing difficulty; Circulation / Coma / Convulsion / Confusion; Dehydration; and Other). Priorities were defined as patients with the 3TPR-MOB priority signs (i.e. Tiny baby (sick child of under 2 months of age); Temperature (child is very hot); Trauma or other urgent surgical condition; Pallor (severe); Poisoning; Pain (severe); Respiratory distress; Restless, lethargy or continuously irritable; Referral; Malnutrition (severe wasting); Oedema of both feet; and Burns) [52].	[50, 51]
3	Estimated proportion of emergency patients who received at least one appropriate treatment	Numerator: Number of emergency patients who received at least one treatment prescribed according to ETAT standards where the standards were reported in Kinoti et al. (manuscript under review in PlosOne). For emergency patients who were prescribed treatment and data on drug availability were missing, we applied the “in-stock” rate for patients with those data. Denominator: Number of outpatients classified as emergency or an emergency sign was noted in the triage section of the form	[50, 51]
Case management of fever and malaria			
4	Proportion of malaria suspects with a malaria test result recorded	Numerator: Number of malaria suspects with a result for a laboratory test or rapid diagnostic test for malaria, where the definition of a malaria suspect was reported in Mbonye et al. [37]. Denominator: Number of malaria suspects	[16, 39, 53]
5	Estimated proportion of malaria cases who received an appropriate antimalarial	Numerator: Number of outpatients treated with appropriate anti-malarial(s), where appropriate antimalarial treatments were quinine and four artemisinin-based combination therapies reported in Mbonye et al. [37]. For patients who were prescribed an antimalarial and data on drug availability were missing, we applied the “in-stock” rate for patients with those data. Denominator: Number of outpatients treated for malaria	[16, 39, 53]
6	Proportion of patients with a negative malaria test result who were prescribed an antimalarial	Numerator: Number of patients with a negative malaria test result prescribed any antimalarial including appropriate treatments and those that do not comply with Ugandan national guidelines. Denominator: Number of patients with a negative malaria test result	[16, 39, 53]
7	Proportion of patients with a positive malaria test result who were prescribed an antibiotic	Numerator: Number of patients with a positive malaria test result prescribed any antibiotic(s), where antibiotic treatment refers to the 31 drugs listed in Mbonye et al. [37]. Denominator: Number of patients with a positive malaria test result	[16, 39, 53]
Case management of respiratory illness			
8	Proportion of pneumonia suspects aged under 5 years assessed for pneumonia	Numerator: Number of child pneumonia suspects with at least one of the three following assessment results recorded: 1) abnormal chest sounds, 2) chest in-drawing, and 3) rapid breaths per minute. A pneumonia suspect was defined as any child aged under five years presenting with cough or who received a diagnosis of “pneumonia” or “cough/cold no pneumonia”. Denominator: Number of child pneumonia suspects. Note: The definition of suspect focused on children with cough; difficulty in breathing was inadvertently omitted from the form.	[54]
9	Estimated proportion of patients aged under 5 years diagnosed with pneumonia who received appropriate antibiotic treatment	Numerator: Number of children diagnosed with pneumonia treated with appropriate antibiotic, where appropriate antibiotic treatment referred to six drugs on the revised Medical Form 5: amoxicillin, benzyl penicillin, erythromycin, chloramphenicol, gentamicin, cotrimoxazole, and 11 other drugs that were specified: ampicillin, azithromycin, cefixime, ceftriaxone, cefuroxime, co-amoxiclav, gatifloxacin, levofloxacin, penicillin, phenoxymethylpenicillin, ampiclox (amoxicillin and cloxacillin). For patients who were prescribed an antibiotic and data on drug availability were missing, we applied the “in-stock” rate for patients with those data. Denominator: Number of children diagnosed with pneumonia	[54]
10	Proportion of TB suspects with a first Acid-Fast Bacilli (AFB) smear result	Numerator: Number of TB suspects who get a first AFB smear result, where TB suspect was defined as anyone with a history of: cough for longer than two weeks, cough for less than two weeks and night sweats, cough for less than two weeks and weight loss, TB test ordered, new TB diagnosis, started on initial TB treatment, or referred for TB treatment. For children, the definition extended to anyone who had contact with someone with TB. Denominator: Number of TB suspects. Note: The definition of TB suspect is from the Intensified Case Finding Form for People Living with HIV, contacts of smear positive patients, and HIV care settings [55]. Increasing AFB smears among TB suspects will increase case detection, but this is not the case detection indicator used by the Stop TB program [56].	[52, 56–58]
11	Estimated proportion of patients with AFB smear negative results who received empiric treatment for acute respiratory infection	Numerator: Number of people with AFB smear negative test results who received empiric treatment for acute respiratory infection, including amoxicillin, doxycycline, or erythromycin. For patients who were prescribed an antibiotic and data on drug availability were missing, we applied the “in-stock” rate for patients with those data. Denominator: Number of people with AFB smear negative result	[52, 56–58]
HIV testing and prevention			
16	Proportion of patients with an HIV test result recorded	Numerator: Number of outpatients who were not TB suspects with an HIV test result recorded. TB suspect is defined for Indicator 10. Denominator: Number of outpatients who were not TB suspects. Note: This indicator included anyone who said they knew their HIV status in the protocol, and was revised to comply with the MOH definition based strictly on a laboratory test result on the day of the outpatient visit.	[58]
		Numerator: Number of TB suspects with an HIV test result recorded. TB suspect is defined for Indicator 10. Denominator: Number of TB suspects. Note: This indicator included anyone who said they knew their HIV status in the protocol, and was revised as described above.	

*Abbreviations*: *AFB* Acid-fast bacilli, *CQI* Continuous Quality Improvement, *HIV* Human Immunodeficiency Syndrome, *MOH* Ministry or Health

Among those 12 indicators, Weaver et al.’s reported that IMID alone was associated with statistically significant pre/post improvements in three indicators: outpatients triaged (Indicator 1), emergency and priority patients who were admitted, detained or referred (Indicator 2), and pneumonia suspects under age five assessed for pneumonia (Indicator 8). A combination of IMID and OSS was associated with statistically significant improvements in four indicators: outpatients triaged (Indicator 1), emergency and priority patients who were admitted, detained or referred (Indicator 2), 3) estimated malaria cases who received appropriate antimalarial treatment (Indicator 5), and patients with a negative malaria test result who were prescribed an antimalarial (Indicator 6), for which a decrease was an improvement. The incremental effect of OSS was not statistically significant for any of the 12 indicators, but many of the effect sizes were large.

We hypothesized that OSS improved facility performance more among the providers who attended IMID training (IMID-MLP) compared to the ones who did not (No-IMID). Weaver et al.*’s* analysis included all patients, and we suspected that the No-IMID subgroup could have diluted the effects of OSS in the IMID-MLP subgroup. We therefore sought to test the effect of OSS on the 12 indicators based on the data surveillance system in the two subgroups of health providers: IMID-MLP and No-IMID. The analysis was limited to only outpatient visits with data to identify provider cadres, and whether or not the provider attended IMID training. In this article, the term “indicator” refers to the facility performance indicators.

Also, during the ethical review of the IDCAP protocol, the School of Medicine Ethics and Research Committee of Makerere University expressed concern that the interventions would affect the distribution of the workload. The data surveillance system data provided an opportunity to examine whether workload increased among the IMID-MLP with OSS, and among the No-IMID health workers with OSS. We therefore tested the effect of OSS on changes in workload in each subgroup of providers.

## Methods

### Study design

**Fig. 1:**
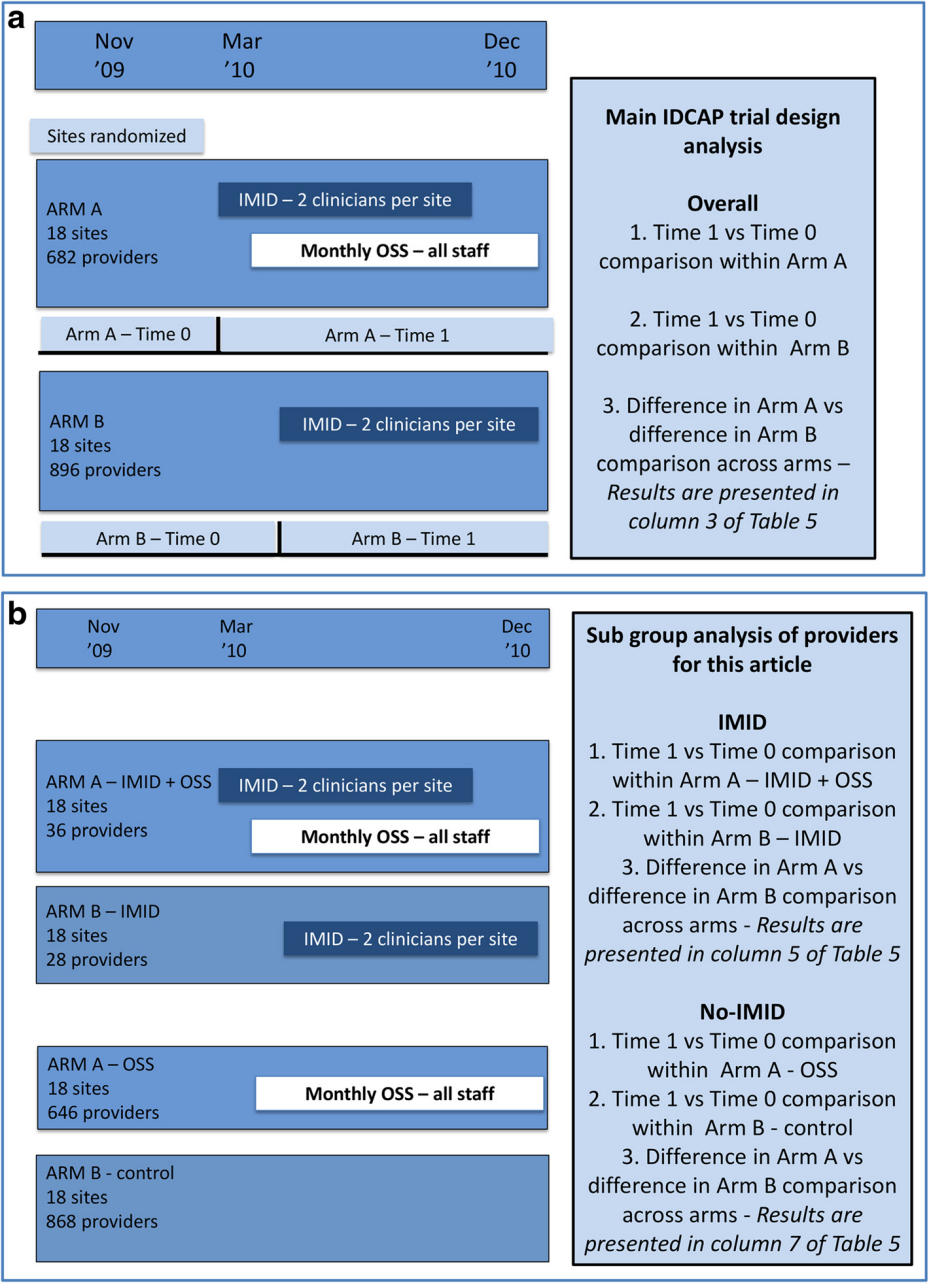
**a** Design of the overall comparison across arms. OSS refers to On-Site Support, IMID to Integrated Management of Infectious Disease, and MLP to mid-level practitioners. **b** Design of the across arm comparisons within the IMID-MLP and No-IMID subgroups

We conducted a secondary analysis of IDCAP data. As noted above, IDCAP was a mixed design study consisting of pre/post and cluster randomized trial components. It was implemented at 36 health center IVs (HCIV) or comparable health facilities across Uganda. Eighteen of the 36 health facilities were randomized to Arm A and the other 18 to Arm B. In both arms, two MLP from each of the 36 health facilities received IMID training. From April to December 2010, all providers at Arm A facilities received nine monthly OSS visits while Arm B did not. The pre/post component measured the effects of the combination of IMID training and OSS in Arm A, and IMID training alone in Arm B. The cluster randomized trial component measured the incremental effect of OSS as the difference between the pre/post effects in the two arms. Figure 1a presents the design of the main test of facility performance indicators, which is reported as the overall result below. Note that the overall results presented in Table 5 differ from the results of *Weaver* et al.*’s* analysis, because only outpatient records with data on provider cadre and whether or not the provider attended IMID training were analyzed. Figure 1b presents the design of the subgroup analysis.

The process of randomization, blinding, sample size calculation, and recruitment for the cluster-randomized trial were previously reported in Weaver et al. [33]. A The IDCAP protocol is summarized by Naikoba et al. [20]. The full protocol can also be accessed as supplementary file in both Mbonye et al. [39], Weaver et al. [33] and is available as supporting information to this paper in Protocol 1. The CONSORT checklist for this subgroup analysis is available as Additional file 1.

### Sites, participants, and inclusion criteria

The 36 study health facilities included 31 health center IV (HCIV) and five small hospitals comparable in size to HCIV. A HCIV serves a population of about 100,000 people providing basic preventive and curative care, and is at the highest referral point for a health sub-district [40]. Two MLP, defined as clinical officers, registered nurses or midwives, from each health facility participated in the IMID training. Inclusion criteria for facilities and IMID participants have been described elsewhere [20, 29]. Briefly, the key inclusion criteria for selecting IMID participants was involvement in daily management of patients in the outpatient clinics, and spending over 80 % of the time seeing patients at the participating health facilities [20]. All health workers including IMID participants were invited to participate in the OSS sessions. All outpatients participated as part of the routine healthcare seeking process. For the subgroup analyses, as noted above, only records of outpatient visits with data on the health provider’s cadre and whether or not the health provider attended IMID training were included.

### Interventions

IMID was a three-week core course followed by two one-week booster courses at 12 and 24 weeks after the core course and distance learning [29]. The core course was taught in four sessions at the Infectious Diseases Institute in Kampala, Uganda with two sessions for Arm A in March and April 2010 and two sessions for Arm B in May and June 2010.

OSS was a two-day visit to the health facilities in Arm A delivered by mobile teams for nine consecutive months from April to December 2010 [20, 29]. Each mobile team consisted of four members: a medical officer with CQI experience, a clinical officer, a laboratory technologist, and a registered nurse. Each visit focused on one OSS topic as well as follow-up from the previous sessions and was characterized by four distinct activities: the multidisciplinary team training, one-on-one mentorship, breakout sessions, and CQI activities.

### Outcomes

The outcomes were patients per provider used to measure changes in workload, and 12 facility performance indicators based on the data surveillance system whose definitions by numerator and denominator are reported in Table 1, as well as sources of the indicators. Indicator 16, proportion of patients with an HIV test recorded, differs from the earlier report [33], because it excludes registry data from antenatal and tuberculosis clinics.

### Data collection

The data source was the revised Uganda Ministry of Health outpatient medical form (MF5) [41]. Using the MF5, health workers prospectively collected data on every outpatient visit from November 2009 to December 2010. From March 2010, data entry assistants were stationed at each health facility to capture these data electronically in an Epi Info® Version 3.2 (U.S. Centers for Disease Control and Prevention, Atlanta, GA) database. At the end of each month, the data entry assistants transmitted electronic data to the Infectious Diseases Institute where data for all health facilities were merged using Microsoft Excel® 2010 (Microsoft Corporation, Redmond, WA, USA), and further cleaned.

### Data analysis

We used frequencies to describe provider, patients, and outcomes across arms, subgroups, and time periods. The effect of OSS was estimated as a difference in pre/post (Time 0/Time 1) changes between Arm A and Arm B. In Arm A, Time 0 was from November 2009 to March 2010 and Time 1 started after the first IMID training in Arm A and was from April to December 2010. In Arm B, Time 0 was from November 2009 to May 2010 and Time 1 started after the first IMID training in Arm B and was from June to December 2010. During Time 1, nine OSS visits were implemented in Arm A.

Using a generalized linear latent and mixed model [42], data on the number of patients per provider were analyzed as continuous dependent variables with main effects for arm, subgroup, time period and their interaction to estimate pre/post differences within each subgroup in each arm and ratios of relative risk (RRR) for the effect of OSS within each subgroup. The unit of analysis was a subgroup-facility-month, and regressions were estimated with clusters for provider and facility with robust standard errors.

The RRR for a given performance indicator was estimated with main effects for arm, time period and their interaction, using data for the total sample and for each subgroup and the generalized linear model with a Poisson family and log link as described earlier [33, 39]. The unit of analysis was facility month, and all regression analyses were clustered on the health facility. To adjust for over dispersion and using Poisson rather than binomial family, the regressions were estimated with robust standard errors. Multiple comparisons were addressed by basing the statistical tests on α = 0.01 and all results were presented with 99 % confidence intervals (CI).

All regression analyses were adjusted for facility type (public or private not-for-profit), facility level (HCIV or small hospital), data entry assistant on-site, Baylor and previous participation in the national HIV CQI program (see description of strata in [33, 39]). Indicators 1–7 and 10–11 were also adjusted for staffing and patient age (<5, > = 5 years), indicators 8–9 for staffing, and indicator 16 for staffing, TB suspect and patient age group (2–14 months, 18 months – 13 years and ≥14 years) to reflect HIV testing guidelines. All data analyses were conducted using Stata® version 12 (StataCorp, College Station, Texas, USA).

### Ethical considerations

The School of Medicine Research and Ethics Committee of Makerere University Kampala (reference number 2009–175) and the Uganda National Council for Science and Technology (reference number HS-722) approved the IDCAP protocol. The University of Washington Human Subjects Division determined that IDCAP did not meet the regulatory definition of research under 45 CFR 46.102 (d). This subgroup analysis was exempt from review by the University of Antwerp ethical review board.

## Results

### Participant flow

**Fig. 2:**
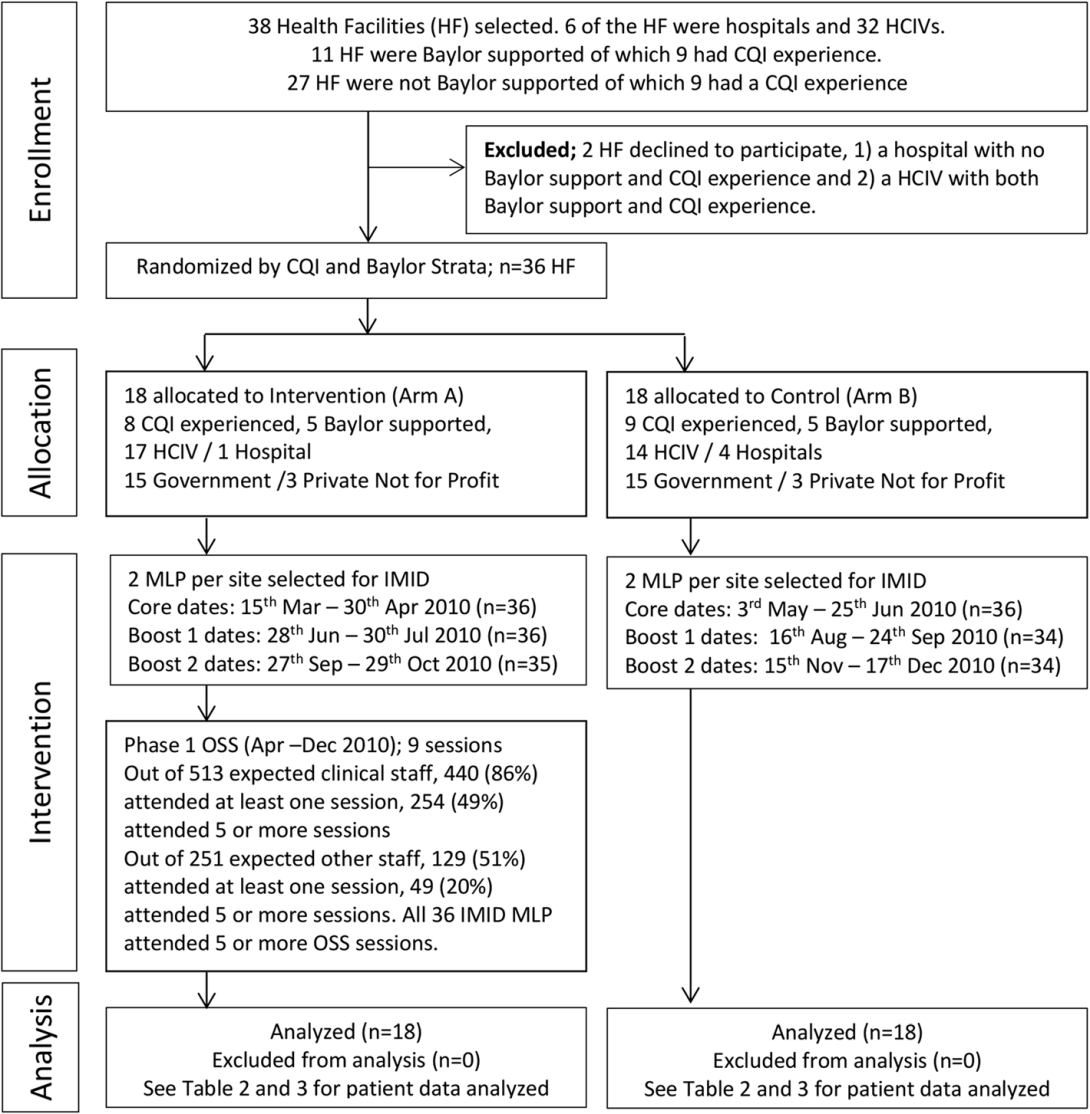
Consort flow diagram – recruitment and randomization

The flow of the participating health facilities, and IMID training and OSS participants is presented in Fig. 2 [33]. All health facilities participated up to the end of the study. Of the 36 individuals who participated in IMID training, one person in Arm A and two in Arm B did not attend one or more boost courses. Attendance of OSS sessions was also not consistent. Out of 513 clinical staff expected to attend multi-disciplinary team sessions, only 440 (86 %) attended at least once; out of 499 clinical staff expected to attend the breakout session, only 344 (69 %) attended at least once; and out of 351 clinical staff expected to attend the clinical mentoring and coaching session, only 186 (53 %) attended at least once. Among those who attended at least one OSS session, the mean number of sessions attended per clinical staff was 4.93 for multi-disciplinary team sessions, 3.64 for breakout sessions and 4.01 for clinical mentoring and coaching sessions.

A total of 777,667 outpatients visited the 36 health facilities during the study period. Of these visits, 669,580 (86.1 %) had data on the provider’s name and cadre, which allowed us to determine whether or not the provider attended IMID training, and were included in this analysis (Table 2). During these visits, patients were seen by 64 IMID-MLP and 1,515 No-IMID providers. Data on 108,087 (13.9 %) visits with missing (91,006), unknown (14,686) or indeterminate (2,395) data on the provider were omitted from the analysis.

**Table 2:** Distribution of providers by arm and subgroup

	Provider	Arm A		Arm B		Total		Patients
		IMID-MLP	No-IMID	IMID-MLP	No-IMID	IMID-MLP	No-IMID	Total (Percent)
1	Medical Officer	0	64	0	80	0	144	32,994 (4.9)
2	Clinical Officer	24	137	19	193	44	330	370,745 (55.4)
3	Nurse	12	172	8	231	20	403	162,542 (24.3)
4	Midwife	0	0	1	0	1	0	51 (0.008)
5	Nursing Assistant	0	110	0	179	0	289	62,968 (9.4)
6	Other less skilled	0	163	0	186	0	349	40,280 (6.2)
	Total	36	646	28	869	64	1,515	669,580

### Baseline

#### Patient volume

In Table 3, baseline data for the number of providers, the number of patients seen, and the estimated number of patients per provider per calendar day is reported by time period, arm and subgroup. The number of patients per provider per calendar day was higher in Arm B with more hospitals than in Arm A in the IMID-MLP subgroup (4.3 vs. 3.7) and similar across arms in the No-IMID subgroup (1.1 vs. 1.1).

**Table 3:** Before and during OSS comparison of average number of patients per provider per month by subgroup and arm

Variable	IMID-MLP		No-IMID	
	Arm A	Arm B	Arm A	Arm B
Part 1: Patient and provider population				
Time 0				
Total number of patients	19,686	25,708	59,614	126,611
Number of providers	35	28	358	559
Number of calendar days	151	212	151	212
Time 1				
Total number of patients	54,904	26,163	174,935	181,956
Number of providers	36	26	496	641
Number of calendar days	275	214	275	214
Part II: Patients per provider per calendar day				
Time 0	3.7	4.3	1.1	1.1
Time 1	5.5	4.7	1.3	1.4
Change (Time 1-Time 0)	1.82	0.37	0.15	0.28
Difference between Arm A and Arm B	1.45		−0.13	
Part III: Regression	aRR (99 % CI), *p*-value	aRR (99 % CI), *p*-value	aRR (99 % CI), *p*-value	aRR (99 % CI), *p*-value
Time 1 – Time 0	1.15 (0.72, 1.85), 0.437	0.96 (0.57, 1.62), 0.828	0.99 (0.76, 1.30), 0.921	1.10 (0.88, 1.37), 0.264
	aRRR (99 % CI), *p*-value		aRRR (99 % CI), *p*-value	
Effect of OSS in each group	1.21 (0.61, 2.38), 0.478		0.90 (0.63, 1.28), 0.443	

^**^Denotes that the effect of OSS was significant at the .01 level and ^*^Denotes that the effect of OSS was significant at the .05 level

The 99% confidence intervals (CI) are based on the .01 level of significance

Abbreviations: *aRR* adjusted relative risk, *aRRR* adjusted ratio of relative risks, *IMID* Integrated Management of Infectious Disease, *OSS* On-site support, *MLP* Mid-level practitioners, *RR* Relative Risk measured the comparisons in pre/post change from Time 0 to Time 1; *RRR* Ratio of Relative Risk Measured the effect of OSS in each subgroup

Estimates were adjusted for: whether the facility received the on-site intervention from Baylor International Pediatric AIDS Initiative or not, facility was implementing continuous quality improvement prior to IDCAP trial, level of health facility (small hospital or health center IV), facility ownership (public or private-not-for profit) and data entry assistant on-site

#### Patient care

In Table 4, baseline data for each indicator are reported by both arm and subgroup. Performance in Arm B was generally higher than in Arm A within both IMID-MLP and No-IMID subgroups for eight out of the 12 indicators. For indicators 6 (Proportion of patients with a negative malaria test result who were prescribed an antimalarial), and 7 (Proportion of patients with a positive malaria test result who were prescribed an antibiotic) a lower percentage indicated better performance, and Arm A had lower percentages than Arm B for both indicators. Performance was highest for indicator 5 (estimated proportion of malaria cases who received an appropriate antimalarial). For the remaining indicators, performance was generally low, did not exceed 64 % in both arms and subgroups, and was as low as 3.1 % for indicator 8 (Proportion of pneumonia suspects aged under 5 years assessed for pneumonia) in the IMID-MLP subgroup of Arm A.

**Table 4:** Frequencies and percentages by arm, time period and subgroup

Ind no.	Indicator	Arm A				Arm B			
		Time 0		Time 1		Time 0		Time 1	
		No-IMID	IMID-MLP	No-IMID	IMID-MLP	No-IMID	IMID-MLP	No-IMID	IMID-MLP
1	Proportion of outpatients triaged	15,463 (27.7 %)	6,104 (32.8 %)	145,205 (84.6 %)	49,611 (92.0 %)	55,052 (45.4 %)	15,894 (63.8 %)	127,500 (71.4 %)	22,722 (87.8 %)
2	Proportion of emergency and priority patients who were admitted, detained or referred	425 (10.1 %)	133 (23.2 %)	4,268 (36.1 %)	1,931 (40.8 %)	2,474 (15.4 %)	909 (28.3 %)	4,251 (25.8 %)	793 (35.2 %)
3	Estimated proportion of emergency patients who received at least one appropriate treatment	544 (23.9 %)	82 (36.2 %)	1,469 (51.5 %)	737 (57.4 %)	1,956 (31.3 %)	411 (43.4 %)	2,357 (41.7 %)	177 (22.5 %)
4	Proportion of malaria suspects with a malaria test result recorded	13,727 (37.3 %)	5,154 (39.4 %)	53,764 (51.0 %)	16,698 (51.5 %)	24,320 (30.5 %)	5,958 (37.5 %)	38,615 (33.4 %)	6,323 (41.3 %)
5	Estimated proportion of malaria cases who received an appropriate antimalarial	25,438 (83.2 %)	8,650 (77.3 %)	70,090 (92.5 %)	19,463 (93.8 %)	59,950 (87.5 %)	11,624 (88.8 %)	81,959 (85.7 %)	9,363 (82.7 %)
6	Proportion of patients with a negative malaria test result who were prescribed an antimalarial	2,904 (42.4 %)	1,475 (56.6 %)	9,525 (31.9 %)	2,308 (23.2 %)	6,699 (51.9 %)	1,573 (45.7 %)	11,976 (50.4 %)	1,331 (35.6 %)
7	Proportion of patients with a positive malaria test result who were prescribed an antibiotic	3,052 (44.4 %)	1,192 (46.8 %)	10,681 (44.6 %)	3,107 (46.0 %)	5,253 (46.1 %)	1,067 (42.4 %)	7,629 (51.4 %)	965 (37.3 %)
8	Proportion of pneumonia suspects aged under 5 years assessed for pneumonia	343 (3.3 %)	214 (3.1 %)	5,000 (15.4 %)	4,172 (17.6 %)	1,274 (5.7 %)	970 (8.6 %)	5,984 (20.4 %)	3,665 (25.5 %)
9	Estimated proportion of patients aged under 5 years diagnosed with pneumonia who received appropriate antibiotic treatment	634 (50.7 %)	464 (51.5 %)	2,535 (59.3 %)	1,771 (53.8 %)	2,064 (54.7 %)	1,190 (54.1 %)	2,996 (62.9 %)	1,572 (56.2 %)
10	Proportion of TB suspects with a first Acid-Fast Bacilli (AFB) smear result	283 (7.6 %)	220 (7.7 %)	1,342 (8.7 %)	1,100 (8.9 %)	441 (8.9 %)	283 (13.1 %)	884 (13.8 %)	460 (16.4 %)
11	Estimated proportion of patients with AFB smear negative results who received empiric treatment for acute respiratory infection	50 (17.7 %)	33 (17.5 %)	379 (29.8 %)	322 (32.5 %)	123 (21.9 %)	69 (27.9 %)	231 (26.3 %)	102 (24.9 %)
16	Proportion of patients with an HIV test result recorded (all patients ≥2 months)	5,483 (5.6 %)	1,750 (5.6 %)	23,352 (7.7 %)	11,044 (11.4 %)	7,399 (3.4 %)	1,857 (4.4 %)	15,637 (4.8 %)	2,321 (5.2 %)

### Outcomes

#### Patient volume

As shown in Table 3, pre/post change in the number of patients per provider per month from Time 0 to Time 1 among the IMID-MLP subgroup was higher in the Arm A (adjusted relative risk (aRR) = 1.15, 99 % CI = 0.72-1.85, *p* = 0.437) than in Arm B (aRR = 0.96, 99 % CI = 0.57-1.62, *p* = 0.828), and the effect of OSS in this subgroup was not statistically significant (adjusted RRR (aRRR) = 1.21; 99 % CI = 0.61-2.38, *p* = 0.478). In the No-IMID subgroup however, pre/post change was lower in Arm A (aRR = 0.99, 99 % CI = 0.76-1.30, *p* = 0.922) than Arm B (aRR = 0.1.10, 99 % CI = 0.88-1.37, *p* = 0.264), and the effect of OSS was also not statistically significant (aRR = 0.90; 99 % CI = 0.63-1.28, *p* = 0.443).

#### Facility performance

The effect of OSS on 12 facility performance indicators is reported in Table 5. Overall, the incremental effect of OSS was statistically significant for three indicators; increases in the estimated proportion of emergency patients who received at least one appropriate treatment (Indicator 3, aRRR = 2.00; 99 % CI = 1.11-3.79, *p* = 0.003), and estimated proportion of malaria cases who received an appropriate antimalarial (Indicator 5, aRRR = 1.15, 99 % CI = 1.01-1.32, *p* = 0.006), and decrease in proportion of patients with a negative malaria test result prescribed an antimalarial (Indicator 6, aRRR = 0.65, 99 % CI = 0.44-0.98, *p* = 0.006). Within the IMID-MLP, the incremental effect of OSS was statistically significant for three indicators; increases in estimated proportion of malaria cases who received an appropriate antimalarial (Indicator 5, aRRR = 1.26, 99 % CI = 1.02-1.56, *p* = 0.005) and estimated proportion of patients with acid-fast bacilli (AFB) smear negative results who received empiric treatment for acute respiratory infection (indicator 11, aRRR = 2.04, 99 % CI = 1.06-3.94, *p* = 0.005) and decrease in proportion of patients with a negative malaria test result prescribed an antimalarial (Indicator 6, aRRR = 0.49, 99 % CI = 0.26-0.92, *p* = 0.004). Within the No-IMID subgroup, the incremental effect of OSS was statistically significant for only two indicators; increases in the proportion of emergency and priority patients admitted, detained or referred (Indicator 2, aRRR = 2.12, 99 % CI = 1.05-4.28, *p* = 0.006), and estimated proportion of emergency patients who received at least one appropriate treatment (Indicator 3, aRRR = 1.98, 99 % CI = 1.21-3.24, *p* < 0.001). Compared to the No-IMID subgroup, the effect sizes were larger in the IMID-MLP subgroup in seven (3, 5, 6, 8, 10, 11 and 16) of the 12 indicators measured.

**Table 5:** Effect of OSS training on facility performance in each of the IMID-MLP and No-IMID groups

Ind no.	Indicator	Overall (*n* = 669,580)		No-IMID (*n* = 543,119)		IMID-MLP (*n* = 126,461)	
		aRRR (99 % CI)	*p*-value	aRRR (99 % CI)	*p*-value	aRRR (99 % CI)	*p*-value
1	Proportion of outpatients triaged	*1.67 (0.88, 3.16)	0.04	*1.68 (0.90, 3.16)	0.03	1.65 (0.63, 4.31)	0.18
2	Proportion of emergency and priority patients who were admitted, detained or referred	*2.00 (0.93, 4.27)	0.02	**2.12 (1.05, 4.28)	0.006	1.31 (0.63, 2.72)	0.34
3	Estimated proportion of emergency patients who received at least one appropriate treatment	**2.00 (1.11, 3.79)	0.003	**1.98 (1.21, 3.24)	0.000	*2.15 (0.83, 5.54)	0.04
4	Proportion of malaria suspects with a malaria test result recorded	1.21 (0.86, 1.69)	0.15	1.21 (0.81, 1.82)	0.22	1.21 (0.80, 1.81)	0.24
5	Estimated proportion of malaria cases who received an appropriate antimalarial	**1.15 (1.01, 1.32)	0.006	*1.12 (0.99, 1.27)	0.011	**1.26 (1.02, 1.56)	0.005
6	Proportion of patients with a negative malaria test result who were prescribed an antimalarial	**0.65 (0.44, 0.98)	0.006	0.75 (0.48, 1.18)	0.10	**0.49 (0.26, 0.92)	0.004
7	Proportion of patients with a positive malaria test result who were prescribed an antibiotic	0.94 (0.78, 1.13)	0.37	0.92 (0.77, 1.09)	0.20	1.13 (0.66, 1.96)	0.55
8	Proportion of pneumonia suspects aged under 5 years assessed for pneumonia	1.05 (0.35, 3.15)	0.90	0.94 (0.32, 2.78)	0.89	1.21 (0.37, 4.03)	0.68
9	Estimated proportion of patients aged under 5 years diagnosed with pneumonia who received appropriate antibiotic treatment	0.95 (0.57, 1.58)	0.80	0.96 (0.59, 1.15)	0.83	0.95 (0.54, 1.69)	0.84
10	Proportion of TB suspects with a first Acid-Fast Bacilli (AFB) smear result	1.03 (0.45, 2.38)	0.92	0.94 (0.39, 2.28)	0.88	1.19 (0.44, 3.17)	0.66
11	Estimated proportion of patients with AFB smear negative results who received empiric treatment for acute respiratory infection	*1.76 (0.93, 3.32)	0.023	1.58 (0.82, 3.03)	0.07	**2.04 (1.06, 3.94)	0.005
16	Proportion of patients with an HIV test result recorded (all patients ≥2 months)	1.20 (0.73, 1.96)	0.35	1.10 (0.64, 1.87)	0.66	1.48 (0.84, 2.61)	0.07

^**^Denotes that the effect of OSS was significant at the .01 level and ^*^Denotes that the effect of OSS was significant at the .05 level

The 99% confidence intervals (CI) are based on the .01 level of significance

Abbreviations: *aRRR* adjusted ratio of relative risk, *IMID* Integrated Management of Infectious Disease, *OSS* On-site support, *MLP* Mid-level practitioners, *AFB* Acid-fast bacilli, *HIV* HumanImmunodeficiency Syndrome, *TB* Tuberculosis

*aRRR* adjusted Ratio of Relative Risk measured the effect of OSS in each subgroup

Estimates were adjusted for: whether the facility received the on-site intervention from Baylor International Pediatric AIDS Initiative or not, facility was implementing continuous quality improvement prior to IDCAP trial, level of health facility (small hospital or health center IV), facility ownership (public or private-not-for profit) and data entry assistant on-site

## Discussion

There have been limited rigorous randomized education trials that demonstrate the effects of health worker training approaches on patient care outcomes in the resource constrained world. In this study, we have evaluated the effect of an educational outreach intervention involving nine monthly on-site support (OSS) sessions on health facility performance in two groups of trainees – those who were provided with five weeks of off-site classroom education and those who did not have this opportunity.

The OSS intervention did not significantly increase patient load within the subgroup of health providers who did not participate in the IMID training. Among the mid-level practitioners (MLP) who received both IMID training and OSS, the number of patients per provider per calendar day increased; but the changes in workload attributable to OSS were not statistically significant. The effects of OSS on facility performance indicators were heterogeneous. Patients treated by health providers who attended the IMID training benefited from improved case management of malaria and respiratory infections. Patients treated by providers without the IMID training benefited from improvements in emergency triage, assessment and treatment.

The number of patients per provider per calendar day was much lower than we expected. On average, the health providers with IMID training saw 4.64 patients per calendar day, and the other providers 0.83 indicating that the MLP saw 5 times more patients than other providers. This was expected since MLP involved in daily management of patients in the outpatient clinics and spent over 80 % of their time at the health facility seeing patients were prioritized during the selection process [20]. These results for the MLP with IMID training are consistent with those of the Institute for Health Metrics and Evaluation’s report that showed health providers saw an average of three to five patients per day [43]. These results also indicate that health workers in Uganda treat very few patients per day, but this finding contrasts with other reports in which complaints of heavy workload have been cited among health workers in Uganda [44] and other sub-Saharan Africa countries [45, 46]. The reported number of patients seen per provider is an average across the estimated working days for each of the study time periods. It is possible that providers did not work on all estimated days and thus treated a higher number of patients on days when they worked [47]. In a review of studies in six countries including Uganda, it was found that 35 % of the health care providers were absent during their assigned shifts [48]. Among many factors, the absence usually arises because health workers are on leave, attending trainings off-site, tending to their gardens, or doing work in private clinics to supplement their income [47]. If health workers are only on duty an average of 65 % of the time, then the average number of patients per provider per day would increase by one-third. It is also possible that the number of patients’ visits per day was higher on some days than others. Adding in the fluctuations in patient visits and it possible that some clinicians would manage much higher number of patients on some days. Further analysis is required to determine how the number of patients per day seen by clinicians varies by day and across facilities.

Among the health workers without the IMID training, this analysis showed no statistically significant effect of OSS on changes in the volume of patients they managed. In fact, the number of patients seen per provider per calendar day among these health providers remained the same before and during the OSS. Yet a slight increase in volume of patients seen per provider per calendar day among the MLP with IMID training was observed during OSS, although this increase was not statistically significant. This increase in patient load among IMID trainees coupled with no increase among providers who did not attend IMID training, shows a general increase in patient volume overall during OSS, perhaps pointing to an appreciation of better quality services being offered at the health facilities. Rather than shift tasks and workload among cadres, OSS sought to improve facility performance within each cadre’s scope of practice. This could explain why there was no significant increase in patient volume among health workers without IMID training and decrease among health workers without the IMID training.

Overall, OSS was effective for three of the 12 facility performance indicators in this subgroup analysis, compared to none in the main IDCAP trial results [33]. In this subgroup analysis, only records with complete data on the provider’s cadre and IMID attendance were analyzed unlike in Weaver et al.’s [33] analysis that included all patient records. It is plausible that providers with incomplete data also attended fewer OSS or benefited less from them and diluted the effects. We saw evidence of the no-IMID subgroup diluting the OSS effects of the MLP with IMID especially for indicators 11 (patients with AFB smear negative results who received empiric treatment for acute respiratory infection) and 16 (patients with an HIV test result recorded). On the other hand, evidence of the MLP with IMID diluting the effects of the no-IMID subgroup was observed for indicator 2 (emergency and priority patients who were admitted, detained or referred).

The effects of OSS among the MLP with IMID tended to be statistically significant for indicators that reflected clinical decision-making, such as prescribing antimalarials and antibiotics. In contrast, the effects of OSS among the no-IMID subgroup were for emergency, triage and assessment indicators that required a broader team effort to identify and manage patients appropriately. Although OSS did not seek to change a cadre’s scope of practice, it did seek to empower all providers to triage patients and organize immediate care for emergency and priority patients.

The effects of OSS observed in this analysis were heterogeneous across indicators as well as across subgroups. In the overall sample, the average effect size was 34 % and higher than estimates elsewhere. For instance, reviews of continuous medical education and effect of educational outreach visits on health workers’ training reported median improvements ranging from 6.9 to 13.9 % [19] and from 5.6 to 21 % [49] respectively. The effect sizes for the emergency, triage and assessment and malaria case management indicators were generally larger than the effect sizes for case management of respiratory infections and HIV testing. The indicators for the first two coincide with the first two OSS. The OSS sessions for the latter two were delivered later in the intervention, an indication that perhaps, the expectation of immediate improvements over a shorter period of time may have been unrealistic. There is need for monitoring these indicators over more months to observe whether facility performance will improve over time.

Also future researchers should consider designing their trials for a subgroup analysis. Within resource-limited settings in many sub-Saharan African countries, clinicians and other higher level cadres are the ones usually selected to attend continuous medical education trainings to support them improve the quality of diagnosis and treatment. Allied health workers, nurses and other lower level providers often miss out on such opportunities since they mainly play supporting roles to clinicians in providing patient care. OSS would therefore be a great opportunity to improve capacity of these non-clinician health workers to dispense better quality patient care in the entire patient care process. For clinicians and higher level cadres, OSS would be important in supporting them to apply knowledge and skills in diagnosis and treatment acquired from classroom training.

To our knowledge, the IDCAP trial is one of the first randomized trials that evaluated the effects of an integrated educational outreach and quality improvement intervention in sub-Saharan Africa. The results of this study therefore provide important evidence that could inform capacity building interventions and policies for health workers in resource limited settings.

### Limitations

As noted in *Weaver* et al. [33] sample size calculations for the IDCAP trial were based on comparison across arms and an α = 0.05. Consequently, this subgroup analysis may have been underpowered to detect difference-in-difference at α = 0.01 when comparing effect of OSS on facility performance between arms. OSS sessions were delivered gradually for nine months, and its effect may have been underestimated by measuring it during its implementation rather than after.

### Generalizability

This study was conducted in a sample of sub-district level referral HCIVs or comparable small hospitals that met the inclusion criteria. These results can thus be generalized for similar health facilities in Uganda and other countries, and for indicators for which OSS was effective.

## Conclusions

The MLP with IMID training managed thrice as many patients per day compared to other providers. Out of the 12 facility performance indicators, OSS improved three indicators across all patients and among patients managed by MLP with IMID training, but only two indicators among patients managed by providers without IMID training. These results show that OSS supported MLP who diagnosed and treated patients to apply knowledge from IMID. For other providers, OSS supported team work to manage emergency patients. Since provision of patient care is a team effort with clear roles for every health worker, health workers engaged in clinical diagnosis and treatment may need OSS in addition to classroom training, while the nurses and other providers who play a supporting role to the clinicians will need OSS to effectively contribute more to patient care.

## Additional file

Additional file 1: CONSORT checklist for cluster randomised trials. (DOCx 22 kb)
