# Supplementary material for: Effectiveness of educational outreach in infectious diseases management: a cluster randomized trial in Uganda
Source: BMC Public Health. 2016 Aug 4;16:714. doi: 10.1186/s12889-016-3375-4 (PMC4972969; doi:10.1186/s12889-016-3375-4)
Supplement: Additional file 1: — CONSORT checklist for cluster randomised trials. (DOCx 22 kb) [file 12889_2016_3375_MOESM1_ESM.docx]

**Additional file 1. CONSORT checklist for cluster randomised trials**

| * = addition to CONSORT *Modifications to checklist in italics* | | | |
| --- | --- | --- | --- |
| PAPER SECTION and topic | Item Pages | Descriptor | Reported in section |
| TITLE & ABSTRACT | 1 - 3* | How participants were allocated to interventions (e.g., “random allocation”, “randomised”, or “randomly assigned”), *specifying that allocation was based on clusters* | Title, Abstract |
| INTRODUCTION  Background | 4 - 7* | Scientific background and explanation of rationale, *including the rationale for using a cluster design.* | Background |
| METHODS  Participants | 8* | Eligibility criteria for participants *and clusters* and the settings and locations where the data were collected. | Sites, participants, and inclusion criteria |
| Interventions | 9* | Precise details of the interventions intended for each group, *whether they pertain to the individual level, the cluster level or both,* and how and when they were actually administered. | Study Design, Interventions, Fig 1a and Fig 1b |
| Objectives | 6-8* | Specific objectives and hypotheses, *and whether they pertain to the individual level, the cluster level or both*. | Background |
| Outcomes | 9 -12* | Report clearly defined primary and secondary outcome measures, *whether they pertain to the individual level, the cluster level or both*, and, when applicable, any methods used to enhance the quality of measurements (e.g., multiple observations, training of assessors). | Outcomes, Table 1 |
| Sample size | 7-8* | How *total* sample size was determined *(including method of calculation, number of clusters, cluster size, a coefficient of intracluster correlation (ICC or k), and an indication of its uncertainty*) and, when applicable, explanation of any interim analyses and stopping rules. | Study design, Naikoba et al. [20], Weaver et al [33] |
| Randomisation.  Sequence generation | 7-8* | Method used to generate the random allocation sequence, including details of any restriction (e.g., blocking, stratification, *matching*). | Study design, Naikoba et al. [20], Weaver et al [33] |
| Allocation concealment |  | Method used to implement the random allocation sequence, *specifying that allocation was based on clusters rather than individuals and* clarifying whether the sequence was concealed until interventions were assigned. |  |
| Implementation |  | Who generated the allocation sequence, who enrolled participants, and who assigned participants to their groups. |  |
| Blinding (Masking) | 7-8* | Whether or not participants, those administering the interventions, and those assessing the outcomes were blinded to group assignment. If done, how the success of blinding was evaluated. | Study design, Naikoba et al. [20], Weaver et al [33] |
| Statistical methods | 12-13* | Statistical methods used to compare groups for primary outcome(s) *indicating how clustering was taken into account*; methods for additional analyses, such as subgroup analyses and adjusted analyses. | Data analysis |
| RESULTS  Participant flow | 14 - 15* | Flow of *clusters and* individual participants through each stage (a diagram is strongly recommended). Specifically, for each group report the numbers of *clusters and* participants randomly assigned, receiving intended treatment, completing the study protocol, and analyzed for the primary outcome. Describe protocol deviations from study as planned, together with reasons. | Participant flow |
| Recruitment | 14 | Dates defining the periods of recruitment and follow-up. | Study design, Naikoba et al. [20], Weaver et al [33] |
| Baseline data | 15 - 18* | Baseline information for each group *for the individual and cluster levels as applicable* | Baseline data, Tables 2 – 4 |
| Numbers analyzed | 14 - 18* | Number of *clusters and* participants (denominator) in each group included in each analysis and whether the analysis was by “intention-to-treat”. State the results in absolute numbers when feasible (e.g., 10/20, not 50%). | Participant flow, Figure 2, and Table 2 |
| Outcomes and Estimation | 19 - 21* | For each primary and secondary outcome, a summary of results for each group measures *for the individual or cluster level as applicable*, and the estimated effect size and its precision (e.g., 95% confidence interval) *and a coefficient of intracluster correlation (ICC or k) for each primary outcome.* | Outcomes, Tables 3-5 |
| Ancillary analyses | NA | Address multiplicity by reporting any other analyses performed, including subgroup analyses and adjusted analyses, indicating those pre-specified and those exploratory. | Not applicable |
| Adverse events | NA | All important adverse events or side effects in each intervention group. | Not applicable |
| DISCUSSION  Interpretation | 22 – 26 | Interpretation of the results, taking into account study hypotheses, sources of potential bias or imprecision and the dangers associated with multiplicity of analyses and outcomes. | Discussion |
| Generalisability | 26* | Generalisability (external validity) *to individuals and/or clusters (as relevant)* of the trial findings. | Generalisa-bility |
| Overall evidence | 26 - 27 | General interpretation of the results in the context of current evidence. | Conclusion |
